# Comparison of gene-based rare variant association mapping methods for quantitative traits in a bovine population with complex familial relationships

**DOI:** 10.1186/s12711-016-0238-5

**Published:** 2016-08-17

**Authors:** Qianqian Zhang, Bernt Guldbrandtsen, Mario P. L. Calus, Mogens Sandø Lund, Goutam Sahana

**Affiliations:** 1 Department of Molecular Biology and Genetics, Center for Quantitative Genetics and Genomics, Aarhus University, Tjele, 8830 Denmark; 2 Animal Breeding and Genomics Centre, Wageningen UR Livestock Research, Wageningen, The Netherlands

## Abstract

**Background:**

There is growing interest in the role of rare variants in the variation of complex traits due to increasing evidence that rare variants are associated with quantitative traits. However, association methods that are commonly used for mapping common variants are not effective to map rare variants. Besides, livestock populations have large half-sib families and the occurrence of rare variants may be confounded with family structure, which makes it difficult to disentangle their effects from family mean effects. We compared the power of methods that are commonly applied in human genetics to map rare variants in cattle using whole-genome sequence data and simulated phenotypes. We also studied the power of mapping rare variants using linear mixed models (LMM), which are the method of choice to account for both family relationships and population structure in cattle.

**Results:**

We observed that the power of the LMM approach was low for mapping a rare variant (defined as those that have frequencies lower than 0.01) with a moderate effect (5 to 8 % of phenotypic variance explained by multiple rare variants that vary from 5 to 21 in number) contributing to a QTL with a sample size of 1000. In contrast, across the scenarios studied, statistical methods that are specialized for mapping rare variants increased power regardless of whether multiple rare variants or a single rare variant underlie a QTL. Different methods for combining rare variants in the test single nucleotide polymorphism set resulted in similar power irrespective of the proportion of total genetic variance explained by the QTL. However, when the QTL variance is very small (only 0.1 % of the total genetic variance), these specialized methods for mapping rare variants and LMM generally had no power to map the variants within a gene with sample sizes of 1000 or 5000.

**Conclusions:**

We observed that the methods that combine multiple rare variants within a gene into a meta-variant generally had greater power to map rare variants compared to LMM. Therefore, it is recommended to use rare variant association mapping methods to map rare genetic variants that affect quantitative traits in livestock, such as bovine populations.

**Electronic supplementary material:**

The online version of this article (doi:10.1186/s12711-016-0238-5) contains supplementary material, which is available to authorized users.

## Background

Genome-wide association studies (GWAS) have been successful in identifying common variants that are associated with complex diseases and quantitative traits. However, the common variants that have been identified thus far account for only a small fraction of the estimated heritabilities [1–3]. Theoretical and empirical studies suggest that rare variants (defined as those that have frequencies lower than 0.01), could play a significant role in quantitative trait variation [3, 4]. In addition, studies on several Mendelian diseases indicate that common variants may often have a key role as modifiers of the effects of rarer, more highly penetrant contributors to disease risk in humans [5, 6]. Therefore, the detection and investigation of rare variants should help researchers to further understand the genetic architecture of quantitative traits and may provide new ways to use such rare variants for mapping genes and improving accuracies of genomic prediction.

Rare variants are poorly captured by the commonly used single nucleotide polymorphism (SNP) chips, because SNPs on these chips typically have a much higher minor allele frequency (MAF) than rare variants and, thus, are generally in low linkage disequilibrium (LD) with these. Recent technological advances allow us to study individual genomes at the base-pair resolution [7], including the detection of rare variants. Based on a large number of sequenced individuals (e.g. 1000), the optimal sequencing depth required for variants with a frequency lower than 0.01 is ~27 [8]. Therefore, low-coverage sequencing yields low calling accuracy at rare variant sites, and in addition, deep sequencing a large number of individuals remains economically prohibitive. The alternative is to impute high-density SNPs to whole-genome sequence. However, compared to common variants, rare variants are more often private to a sub-population or to families within a population [9], and thus, imputation accuracy for rare variants is considerably lower than for common variants. It has been shown in cattle that imputation accuracy from lower density SNP panels to whole-genome sequence data drops very quickly when allele frequency is lower than 0.1 [10–12]. Although some imputation algorithms, such as that implemented in the IMPUTE2 software, tend to achieve higher imputation accuracies for rare variants than other algorithms, imputation accuracy remains rather low [10]. Thus, imputation of rare variants remains a challenge, and is currently not sufficiently accurate to study the power of gene-based rare variant mapping. Instead deep re-sequencing of a large number of individuals (i.e. at least 1000) is necessary to identify rare variants, but currently this is economically prohibitive although the cost of whole-genome sequencing is continuously decreasing. An alternative approach to study the power to detect rare variants is to carry out a simulation study. Besides, simulated data has the advantage that the causal variants and their simulated effects are known with certainty and therefore, it is possible to compare methods for their accuracies of estimated effects. It is important that the genetic variation of the simulated dataset represents the complete spectrum of allele frequencies and retains the same haplotype structure as the empirical data [13]. Therefore, we used imputed sequence variants for a large number of SNP-array genotyped individuals to compare gene-based rare variant mapping approaches in cattle. Since the phenotypes were simulated based on imputed sequence variants, imputation errors did not distort the individuals’ phenotypes.

Methods for GWAS based on common SNP variants are well established [3]. However, mapping rare variants remains a challenge and rare-variant association studies are generally “gene-based”, in the sense that rare variants that are located within the same gene are grouped and then statistical methods are applied to assess the significance of the association between the phenotype and the combined rare variants. Cirulli [14] emphasized the increasing importance of gene-based analyses in a review of 150 exome sequencing studies that claim that a disease can be caused by different rare variants in the same gene. Recently, guidelines on how to combine rare variants in gene-based analyses were formulated by MacArthur et al. [15].

Several classes of statistical methods have been developed for the analysis of rare variants for ‘case–control’ designs and quantitative traits in humans for both randomly sampled and related individuals [16–19]. A short overview of the approaches is given below.

One broad class of such methods is known as the “burden test” [16, 18, 20, 21]. A burden test collapses multiple rare variants in a region of the genome into a single meta-allele to represent a genetic burden score. These meta-alleles are then used in association analyses. The power of these burden tests depends on the effect of the pooled variants and assumes that the effects of the rare alleles at different variant sites in a region of interest are in the same direction. Recent developments around these burden tests have enabled the analysis of data on related individuals [22, 23].

The second broad class of methods comprises variance component tests, such as that implemented in the C-alpha [17] and sequence kernel association test (SKAT) [19]. Variance component tests aggregate individual variant statistics that measure the similarity of the variants within a region and incorporate flexible weights to boost the power of the analysis. Compared to the burden test, variance component tests are more robust for the identification of a gene even when multiple rare variants within the targeted gene have effects in different directions (positive and negative). There are also extensions for this kind of method for related individuals such as that implemented in famSKAT [22] and other similar approaches [23–25].

The third category of methods combines burden tests and variance component tests to exploit the strengths of both approaches. This is implemented in the software SKAT-O for unrelated individuals [26] and in MONSTER (minimum *p* value optimized nuisance parameter score test extended to relatives) for related individuals [27]. These methods introduce a nuisance parameter that defines the trade-off between burden tests and variance component tests, and is adaptively determined from the data to optimize power. Therefore, the combination of these two tests will be optimally balanced by the data itself and can detect both the common effect across rare variants (as in the burden tests) and the individual deviations from the average effect (as in the variance component tests).

Several studies have mapped rare variants that contribute to complex diseases in humans by using deep exome sequencing [9, 15, 28, 29]. However, to date, association studies for rare variants in cattle and other livestock species have not been reported. Increasing access to a large number of whole-genome sequences [30] and availability of exome sequence data in the near future could be used to map rare variants in cattle. This will open new opportunities to capture rare variants that affect economic traits in cattle especially those that are related to disease susceptibility, which, so far, was not possible by using SNP chip data. This should substantially improve success both in finding causative mutations and using the information for genomic selection to improve accuracy of prediction. Once the causative mutations are identified for one population, they can be directly tested in other populations and thus, results may be transposable from one breed to another.

The above-mentioned methods for rare variant association mapping were developed for human studies for which samples are obtained at random from a population or data that originate from small families, e.g. trio and sib-pair analyses. In contrast, bovine datasets usually include large half-sib families, and intensive artificial selection in cattle may pose special issues that are related to data analysis. For example, rare variants may be confounded with family structure, making it more difficult to disentangle their effects from family mean effects. In addition, the availability of large half-sib family sizes in cattle has the advantage that rare variants may be observed at a higher frequency within extended families compared to the population as a whole. The suitability of the above-described statistical methods that were developed to map rare variants for quantitative traits in humans still remains unexplored for data structures such as those of cattle and other livestock species. Thus, the objective of our study was to investigate power and type I errors of several approaches used to map rare variants in bovine data. Our hypothesis is that the power of the specialized methods that were developed to detect rare variants in the human genome will be higher than that of a linear mixed model approach, which is currently the method of choice to map common variants in the bovine genome. Thus, we propose method(s) for rare variant mapping in livestock populations, which should contribute to the development of models that are geared towards exploiting rare variants in genome-assisted breeding.

## Methods

### Statistical methods

#### Statistical methods for rare variant mapping

The statistical methods that we tested for rare variant mapping were famBT [22], famSKAT [22] and MONSTER [27]. The famBT method is a burden test that accounts for family relationships and assumes that the effects of all the rare variants are in the same direction [22] while the family-based SKAT (famSKAT) method makes no assumption on the direction of the effects of rare variants [22]. The MONSTER method adaptively determines a nuisance parameter to adjust to the unknown composition of the effects at rare variant sites by applying a mixed effects model that accounts for covariates and additive polygenic effects [27].

When written in more conventional animal breeding notation, the MONSTER model becomes:

$${\mathbf{y}} = {\mathbf{X}}{\varvec{\upgamma}} + {\mathbf{M}}{\varvec{\upbeta}} + {\mathbf{Zu}} + {\mathbf{e}},$$

where **y** is a vector of phenotypes, **X** is a design matrix for fixed covariates including the intercept, **γ** is a vector of unknown covariate effects, **Z** is an incidence matrix relating phenotypes to the corresponding random polygenic effect, **u** is a vector of random polygenic effects that follows a multivariate normal distribution $N(\mathbf{0},{\mathbf{A}}\sigma_{a}^{2}$), where **A** is the additive genetic relationship matrix and $\sigma_{a}^{2}$ is the polygenic variance, **e** is a vector of random residuals, ${\mathbf{e}} \sim N\left( {\mathbf{0},{\mathbf{I}}\sigma_{e}^{2} } \right)$, **M** is a *n* × *m* matrix that encodes the genotype at the *m* tested variant loci and *n* is the number of individuals with *m*_*ij*_ representing the allele dosage (0, 1 or 2) of the minor allele at the *j*-th variant of individual *i*, and **β** is a vector of (possibly correlated) random effects of the *m* variants, ${\varvec{\upbeta}} \sim N\left( {\mathbf{0},{\mathbf{R}}_{\rho } \sigma_{q}^{2} } \right)$, ${\mathbf{R}}_{\rho } = \left( {1 - \rho } \right){\mathbf{I}} + \rho {\mathbf{I}}$ with $0 \le \rho \le 1$. The limiting cases *ρ* = 0 and *ρ* = 1 correspond to models famSKAT and famBT, respectively. This method for detecting rare variants is referred to as MONSTER [27]. A grid of 11 equally-spaced points: values of *ρ* i.e. $\rho_{1} = 0, \rho_{2} = 0.1, \ldots , \rho_{10} = 0.9, \rho_{11} = 1$ were tested in MONSTER. When *ρ* = 0, MONSTER is equivalent to famSKAT and when *ρ* = 1, MONSTER is equivalent to famBT.

To detect associations between a trait and a genomic region of interest, we tested the null hypothesis *H*_0_ that $\sigma_{q}^{2} = 0$ against *H*_1_ that $\sigma_{q}^{2} > 0$. This analysis was done using the software MONSTER [27]. To access the type I error rate, the null model was tested for 1000 replicates for which the effects for all rare variants were assumed to be equal to 0. The genomic control coefficient λ [31], which for test statistics measures the departure of the median *p* value from its expectation under the null hypothesis, was calculated for all statistical methods considered to detect rare variants.

#### Statistical methods for GWAS with common variants

We compared MONSTER, famBT and famSKAT to two methods that are used for association mapping of common variants: a linear mixed model [32] and a simplified linear mixed model as implemented in the EMMAX software [33]. These methods were included to investigate their ability to map rare variants and are briefly described below.

The linear mixed model (LMM) carries out a SNP-by-SNP analysis. Complex familial relationships are the primary confounding factor in GWAS of livestock populations. In cattle, LMM, which model the effects of relationships among individuals through polygenic effects, can control the false positive rate caused by family structure and population stratification [34, 35]. Here for the LMM, association between a SNP and a phenotype was assessed by a single-locus regression analysis using the following equation:

$${\mathbf{y}} = \mathbf{1}^{\prime}\mu + {\mathbf{m}}g + {\mathbf{Zu}} + {\mathbf{e}},$$

where **y** is the vector of phenotypes, **1** is a vector of ones, *μ* is the general mean, **m** is a vector of allele dosages (ranging from 0 to 2) that associate records to the marker effect, *g* is the scalar additive effect of the SNP, **Z** is an incidence matrix relating phenotypes to the corresponding random polygenic effect, **u** is a vector of random polygenic effects that follows a multivariate normal distribution $N\left( {\mathbf{0},{\mathbf{A}\sigma}_{a}^{2} } \right)$, where **A** is an additive relationship matrix and *σ*_*a*_^2^ is the polygenic variance, and **e** is a vector of random environmental deviates that follows a normal distribution $N\left( {\mathbf{0},{\mathbf{I}\sigma}_{e}^{2} } \right)$, where σ_*e*_^2^ is the error variance and **I** is an identity matrix. The model was fitted by restricted maximum likelihood (REML) using the software DMU [36], and the null hypothesis *H*_0_ that *g* = 0 was assessed using a *t*-test. The null hypothesis was tested with 1000 replicates and the results are presented as the null model. The genomic control coefficient [31] was used to correct for stratification by adjusting association statistics at each SNP by the overall inflation factor (λ). A SNP was considered to be significantly associated with a trait if the *p*-value was below a significance threshold after correction for multiple-testing. We used two different multiple-testing correction approaches that are described in section “Comparison of different methods used to map rare variants in the simulation*”*.

Single variant association analysis using a LMM for full sequence variants is computationally demanding, i.e. it requires a computation time of ${\text{O}}\left( {{\text{MN}}^{3} } \right)$, where M is the number of SNPs and N is the number of samples, since variance component estimation is repeated for each candidate SNP [37]. Therefore, association analysis for each imputed sequence variant was also carried out using the efficient mixed-model association (EMMA) approach where the variance components are estimated once instead of for each variant using the EMMAX software [33]. Briefly, the polygenic and error variances are estimated using the following variance component model: ${\mathbf{y}} = 1\mu + {\mathbf{Zu}} + {\mathbf{e}}$, where $Var\left( y \right) = {\mathbf{G}}\sigma_{a}^{2} + {\mathbf{I}}\sigma_{e}^{2}$, *μ* is the intercept, **y** is the vector of phenotypes, **G** is the genomic relationship matrix that is built based on high-density (HD) SNP genotypes, **I** is an identity matrix, $\sigma_{a}^{2}$ is the additive genetic variance and $\sigma_{e}^{2}$ is the error variance. In a second step, the SNP effect is obtained using a generalized linear regression model model:

$${\mathbf{y}} = {\mathbf{1}}\mu + {\mathbf{m}}g + {\varvec{\upeta}},$$

where **m** is a vector of the imputed allele dosages (ranging from 0 to 2), and **η** is a vector of random residual deviates with variance ${\mathbf{G}}\sigma_{a}^{2} + {\mathbf{I}}\sigma_{e}^{2}$.

### Individual genotypes and simulation of phenotypes

In total, the genotypes of 27,119 Holsteins animals were available for this study from the Illumina 54 k SNP array version 1 or 2 (Illumina Inc., San Diego). The number of SNPs remaining after quality control was equal to 43,415, for more details see [38]. However, due to the computational constraints, we limited the analysis by including only the genes on chromosome 10 (arbitrarily picked) and for 5000 randomly selected individuals. The positions of the SNPs on the bovine genome were taken from the UMD3.1 Bovine genome assembly [39]. The 54 k genotypes for chromosome 10 of the 5000 randomly sampled animals together with 22,119 other animals were imputed to whole-genome sequence data using a two-step approach with the IMPUTE2 software [40]. Average kinship between the sampled bulls was equal to 0.0017 and the 5000 sampled bulls were sired by 632 bulls that had 1–136 sons in the dataset, with a mean value of 7.9. The heat map of the relationships between the 5000 sampled individuals is in Additional file 1: Figure S1.

Approaches for rare variant mapping are gene-based, thus the results cannot be easily averaged across multiple genes. Therefore, based on the number of rare variants and level of LD between variant sites, two genes on bovine chromosome 10 were selected for this study: (1) the ENSEMBL Gene ID: *ENSBTAG00000018852* located between 1,116,669 and 1,212,429 bp that comprised 635 annotated SNPs in its transcribed region; the 222 rare variants (with a MAF < 0.01) within this gene were grouped into different SNP sets according to their MAF; the average pairwise LD (r^2^) for these variants was equal to 0.149 with an average distance of 83 bp between variants; and (2) the ENSEMBL Gene ID: *ENSBTAG00000035858* located between 610,854 and 933,224 bp that included 3015 annotated SNPs and 309 rare variants (MAF < 0.01); the average pairwise LD (r^2^) for these variants was equal to 0.74 with an average distance of 106 bp between variants.

Phenotypes were simulated as the sum of three components, i.e. a polygenic effect, a QTL effect computed as the sum of the simulated effects of the underlying rare variants, and a random error. The polygenic effects were simulated based on pedigree records. The effects of rare variants were simulated as random effects. Four scenarios with respect to the MAF of the causal variants were considered. Rare variants were grouped into four classes based on MAF for the sampled individuals i.e.: 0.01 ≤ MAF < 0.02; 0.005 ≤ MAF < 0.01; 0.001 ≤ MAF < 0.005; and MAF < 0.001. Two different approaches for assigning effects to rare variants were followed: within a MAF class either multiple rare variants contributed to the total QTL effect or only one rare variant contributed to the whole QTL variance. In the scenarios with multiple causal variants, half of the rare variants within each MAF class were assigned an effect.

Three levels of heritability for the trait were considered i.e. 0.3, 0.5 and 0.8. In addition, three levels of QTL variances were considered. The variance explained by the QTL (i.e. the collective effect of the causal rare variants within the gene) was equal to 0.1, 0.5 or 1 % of the total genetic variance when the heritability was equal to 0.5. In the scenarios with multiple causal variants, the sum of the variance explained by individual causal variants was set equal to the predefined total QTL variance. The QTL effect (*α*) was then calculated by the following equation and each causal rare variant was assigned an effect with a certain weight (as defined next):

$$\alpha^{2} = \frac{{V_{qtl} }}{{Var\left( {\mathbf{M}} \right)}},$$

where *V*_*qtl*_ is the proportion of genetic variance explained by the QTL multiplied by the total genetic variance (i.e. 0.1, 0.5 and 1 %), **M** is the genotype dosage matrix including the loci which have a QTL effect. The weights were assigned in order to add QTL effects on the simulated phenotypes. Note that this formula considers the genotype variance at the QTL, as well as the co-variance between the QTL, and that it yields one value for *α* that is used for all QTL. Therefore, the total QTL effect for each animal was calculated as:* α***M**.

In addition to the QTL effects, an additive polygenic effect was simulated with a variance component proportional to the kinship matrix. The polygenic effects were sampled from the following normal distribution, proceeding from the oldest to the youngest animal:

- Founder: $$a^{F} \sim N\left( {0,1} \right)$$,
- Offspring with one parent known: $a^{O1} \sim N\left( {\frac{a}{2},\left( {\frac{3}{4} - \frac{F}{4}} \right)\sigma_{a}^{2} } \right)$,
- Offspring with two known parents: $a^{O2} \sim N\left( {\frac{{a_{s} + a_{d} }}{2},\left( {\frac{1}{4}\left( {1 - F_{s} } \right) + \frac{1}{4}\left( {1 - F_{d} } \right)} \right)\sigma_{a}^{2} } \right)$,

where *a*^*F*^ is the polygenic effect for a founder, i.e. an animal with both parents unknown, and *a*^*O*1^ or *a*^*O*2^ are the polygenic effects for animals with one or two known parents, respectively, *a, a*_*s*_ and *a*_*d*_ are the polygenic effects for the known parent, the sire and the dam, respectively, *F,F*_*s*_ and *F*_*d*_ are the inbreeding coefficients for the known parent, the sire and dam, respectively, and $\sigma_{a}^{2}$ is the additive genetic variance.

Finally, an independent error variance component was also simulated to account for measurement error and individual-specific variability $\left( {e \sim N\left( {0,\sigma_{e}^{2} } \right)} \right),$ where $\sigma_{e}^{2}$ is the error variance, which is equal to 20, 50 or 70 % of the phenotypic variance.

### Simulated scenarios

A scenario with a sample size of 1000 individuals, a heritability of 0.5 and a QTL that explained 1 % of the total additive genetic variance was considered as the base scenario and used for comparison with the other scenarios (Table 1). Four MAF classes of rare variants (0.01 ≤ MAF < 0.02; 0.005 ≤ MAF < 0.01; 0.001 ≤ MAF < 0.005; and MAF < 0.001) based on MAF calculated from the whole population (27,119 Holsteins animals) were considered as causal variants for each heritability and QTL variance scenario. Two additional heritability levels (0.3 and 0.8) were simulated to compare with the heritability of the base scenario ($h^{2} = 0.5$). Different proportions (0.1, 0.5 and 1 %) of additive genetic variance explained by the QTL were compared for the scenario with MAF class 0.001 ≤ MAF < 0.005. For low QTL variance scenarios (0.1 and 0.5 %), two sample sizes of 1000 and 5000 randomly selected individuals were compared. One hundred replicates were simulated for each scenario.

**Table 1:** Scenarios used in the simulation

Heritability	MAF	Proportion of additive genetic variance explained by the QTL	Sample size in the test
0.3	0.01 ≤ MAF < 0.02 0.005 ≤ MAF < 0.01 0.001 ≤ MAF < 0.005 MAF < 0.001	0.01	1000
0.5	0.01 ≤ MAF < 0.02 0.005 ≤ MAF < 0.01 0.001 ≤ MAF < 0.005 MAF < 0.001	0.01	1000
0.8	0.01 ≤ MAF < 0.02 0.005 ≤ MAF < 0.01 0.001 ≤ MAF < 0.005 MAF < 0.001	0.01	1000
0.5	0.001 ≤ MAF < 0.005	0.001; 0.005 or 0.01	1000
0.5	0.001 ≤ MAF < 0.005	0.001	1000; 5000
0.5	0.001 ≤ MAF < 0.005	0.005	1000; 5000

### Comparison of methods used to map rare variants in the simulation

To analyze samples of related individuals, three rare variant mapping methods famBT [22], famSKAT [22] and a combination of these two methods (MONSTER) [27] were compared. In addition, linear mixed model approaches as implemented by EMMAX [33] and DMU [36] were used. The kinship matrix used for LMM in DMU was based on the pedigree-based matrix (DMU-AMAT) while for EMMAX both a pedigree-based and a genomic relationship matrix using 50 k genotypes of the individuals computed by the “emmax-kin” option were used (EMMAX_AMAT; EMMAX_GMAT). No prior weights were assigned for any variants in the rare variant mapping of all tested methods.

The power of each method was estimated as the proportion of runs that significantly detected loci that were simulated to be causal. A significance level of 0.05 after Bonferroni correction was used for each scenario. The *p* values should be corrected by the total number of SNP sets tested for the MONSTER, famBT and famSKAT methods (there were five SNP tests: one for common variants (MAF ≥ 0.02) and four SNP sets based on the following MAF classes of rare variants: 0.01 ≤ MAF < 0.02; 0.005 ≤ MAF < 0.01; 0.001 ≤ MAF < 0.005; and MAF < 0.001). Thus, if the *p* value for a tested SNP set with simulated QTL was less than 0.05/5, it was considered to be significant. For EMMAX and DMU, the *p* value for simulated QTL was corrected by the total number of SNPs tested. For example, if the *p*value for the simulated QTL was less than 0.05/635, it should be considered as significant for Gene ID: *ENSBTAG00000018852*. However, all the variants tested here are located within a gene and therefore are not independent because of the LD between them. Therefore, we used an alternative multiple-testing correction method based on calculating the effective number of independent SNPs for total number of SNPs according to [41]. Based on this approach, the effective number of independent SNPs was equal to 17 for Gene ID: *ENSBTAG00000018852* and the corresponding eigenvalues explained 99.5 % of the SNP data variation. Based on these criteria, if the *p* value for the simulated QTL was less than 0.05/17 for the single variant analysis using EMMAX or DMU, it was considered as significant. The standard errors for each scenario were calculated from bootstrapping based on 100 re-samplings from the 100 simulation runs.

## Results

### Comparison of different methods with the null model

**Fig. 1:**
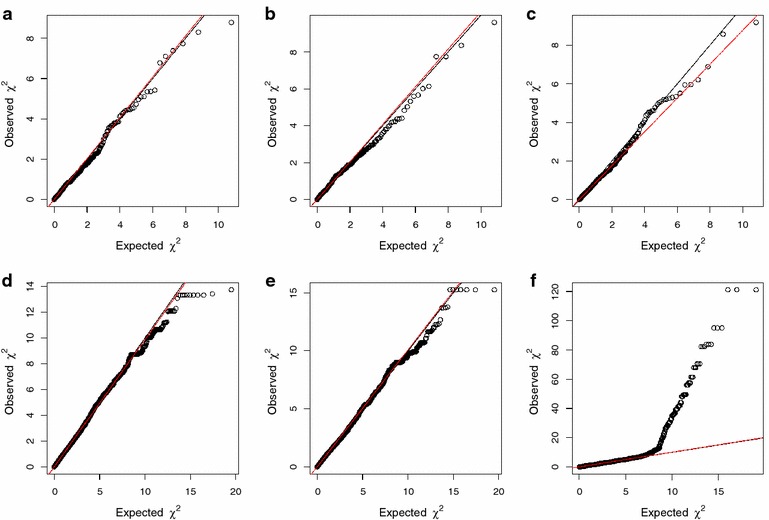
Quantile–quantile plots of the null models with different methods. **a** MONSTER. **b** famBT. **c** famSKAT. **d** EMMAX using the **A** matrix. **e** EMMAX using the **G** matrix. **f** DMU using the **A** matrix

 Figure 1 shows the quantile–quantile plots for the data simulated under the null model (no QTL present). The estimated *λ* (genomic control) values for MONSTER, famBT, famSKAT, EMMAX_AMAT, and EMMAX_GMAT were less than 1, indicating that the *p* values closely followed the expected distribution under the null hypothesis. Therefore, these methods showed no evidence of inflation of the *p* values under the null model. However, some of the observed *χ*^2^ values for DMU_AMAT were far too large, which indicated very high false-positive values (Fig. 1). However, when rare variants with extremely low MAF (MAF < 0.001) were excluded, the estimated *λ* for DMU_AMAT followed the expected distribution under the null hypothesis very well (see Additional file 2: Figure S2). The type I error rate for DMU_AMAT was much higher than that for the other methods (MONSTER, famBT, famSKAT, EMMAX_AMAT, and EMMAX_GMAT) using either Bonferroni correction or multiple-testing correction based on the effective number of independent SNPs (see Additional file 3: Figure S3). However, using the effective number of SNPs to correct the significance level also increased type I error rate for linear mixed models (EMMAX_AMAT, EMMAX_GMAT and DMU_AMAT) (see Additional file 3: Figure S3).

### Comparison of the power of different methods with different scenarios

**Fig. 2:**
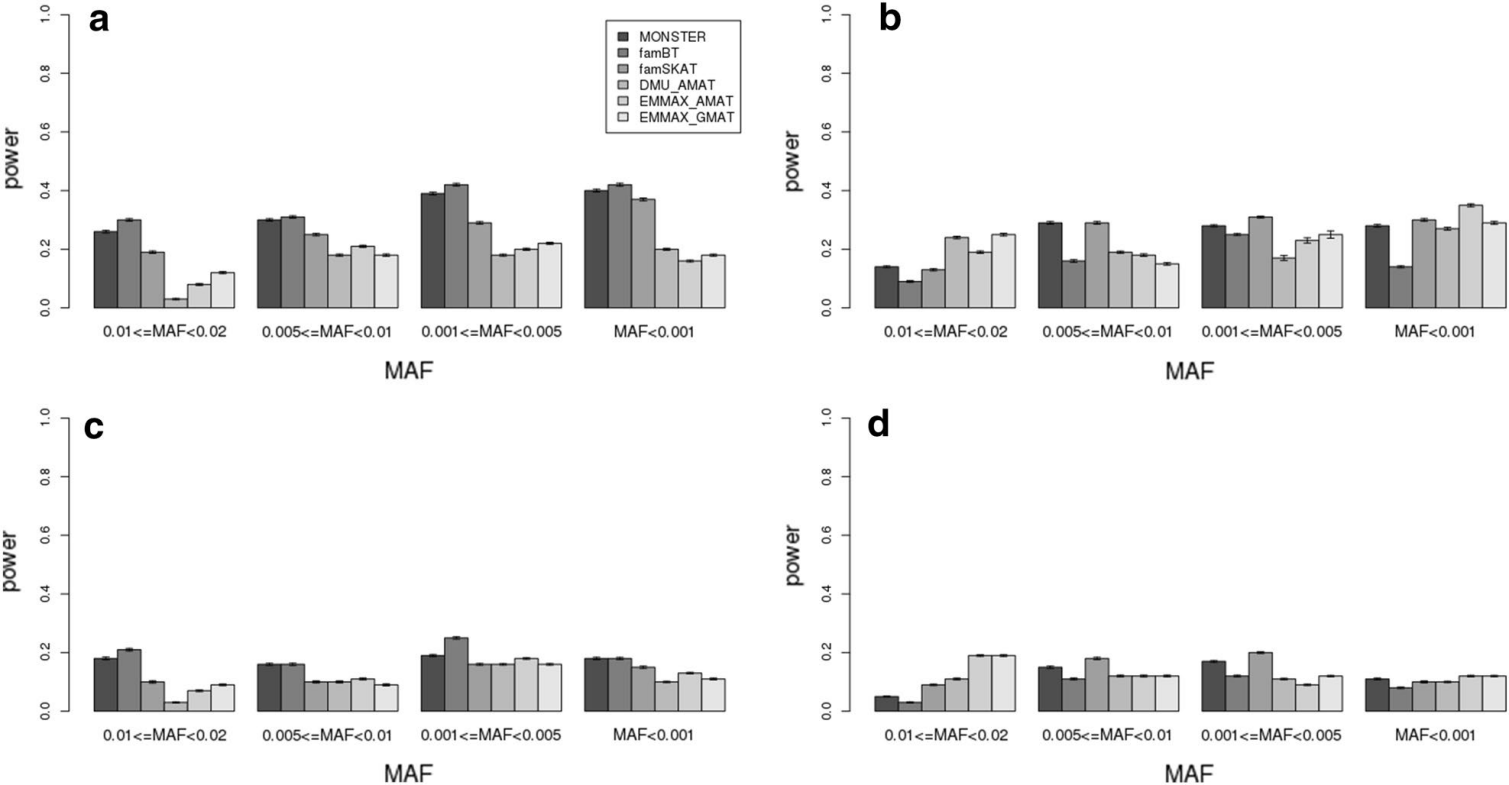
Comparison of the power of rare variant mapping methods in scenarios with different MAF for rare variants and heritabilities. **a**, **b** Heritability = 0.5; 0.01 ≤ MAF < 0.02, 0.005 ≤ MAF < 0.01, 0.001 ≤ MAF < 0.005, MAF < 0.001; proportion of additive genetic variance explained by the QTL = 0.01; sample size in the test = 1000; with multiple rare variants simulated as QTL (**a**) and one rare variant simulated as a QTL (**b**). **c**, **d** Heritability = 0.3; 0.01 ≤ MAF < 0.02, 0.005 ≤ MAF < 0.01, 0.001 ≤ MAF < 0.005, MAF < 0.001; proportion of additive genetic variance explained by the QTL = 0.01; sample size in the test = 1000; with multiple rare variant simulated as a QTL (**c**) and one rare variant simulated as a QTL (**d**)

**Fig. 3:**
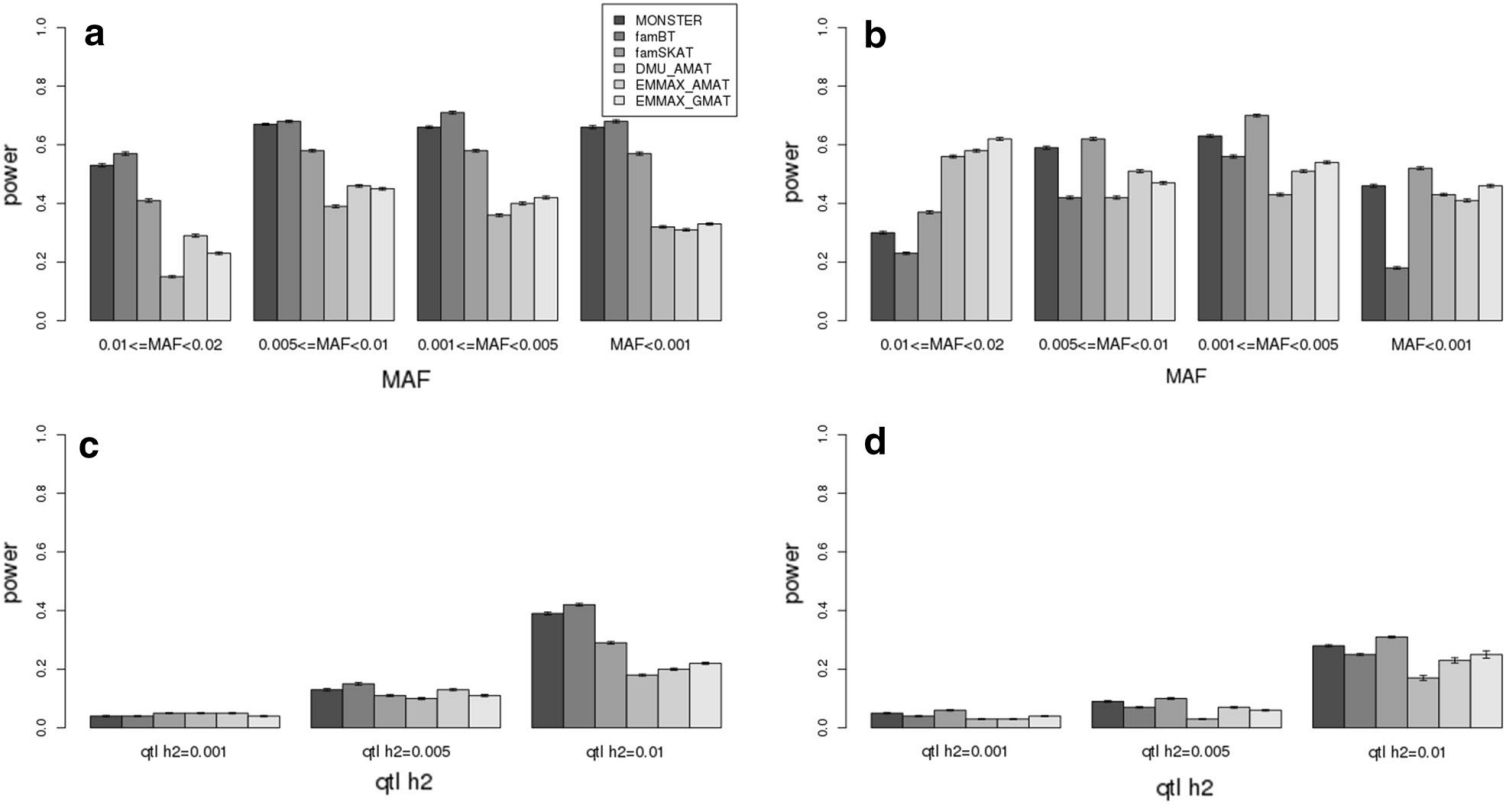
Comparison of the power of rare variant mapping methods in scenarios with different heritabilities and proportions of additive genetic variance explained by the QTL. **a**, **b** Heritability = 0.8; 0.01 ≤ MAF < 0.02, 0.005 ≤ MAF < 0.01, 0.001 ≤ MAF < 0.005, MAF < 0.001; proportion of additive genetic variance explained by the QTL = 0.01; sample size in the test = 1000; with multiple rare variants simulated as QTL (**a**) and one rare variant simulated as a QTL (**b**). **c**, **d** Heritability = 0.5; 0.001 ≤ MAF < 0.005; proportion of additive genetic variance explained by the QTL = 0.01, 0.005, 0.001; sample size in the test = 1000; with multiple rare variant simulated as a QTL (**c**) and one rare variant simulated as a QTL (**d**)

**Fig. 4:**
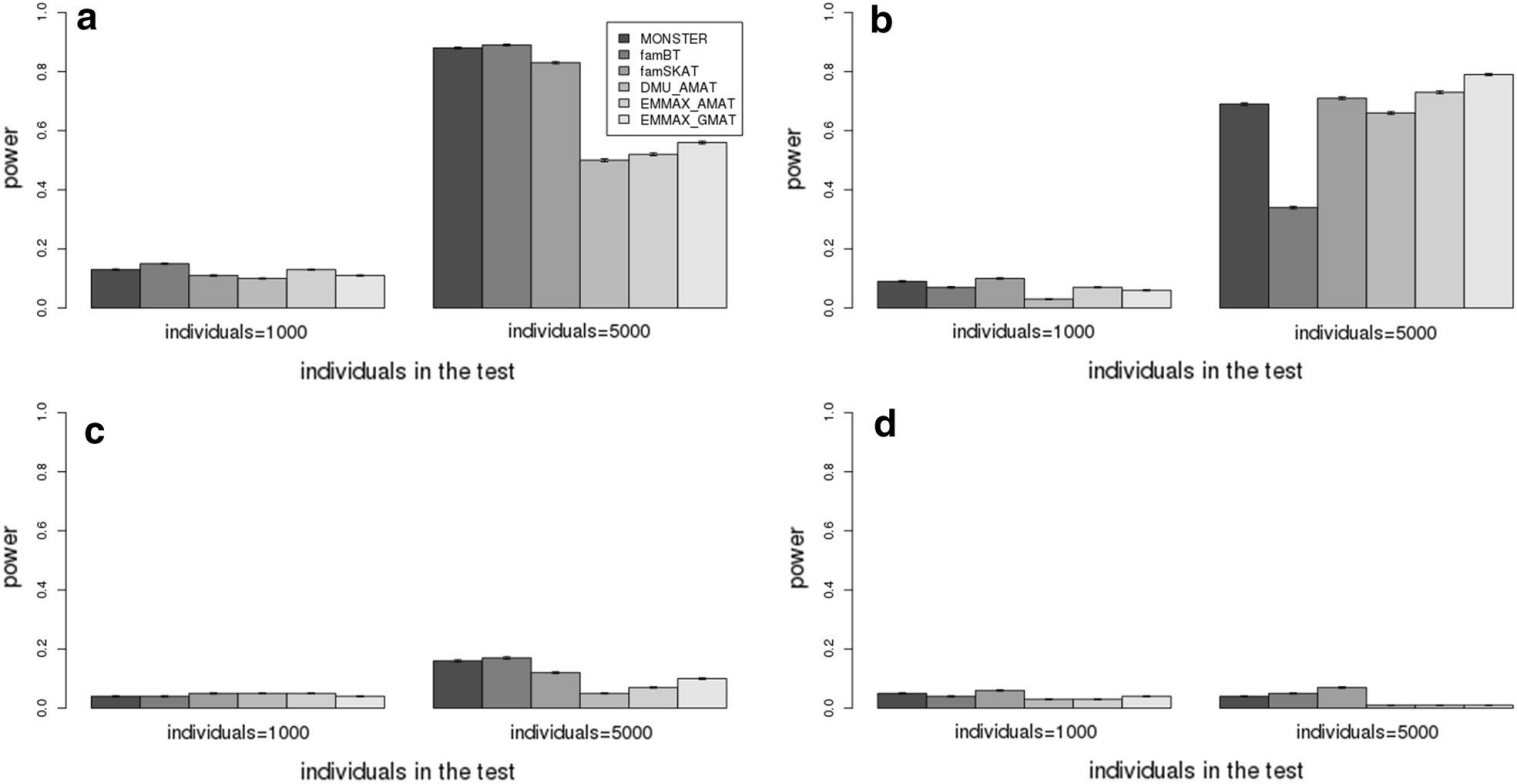
Comparison of the power of different methods in scenarios with different sample sizes. **a**, **b** Heritability = 0.5; 0.001 ≤ MAF < 0.005; proportion of additive genetic variance explained by the QTL = 0.005; sample size in the test = 1000, 5000; with multiple rare variants simulated as QTL (**a**) and one rare variant simulated as QTL (**b**). **c**, **d** Heritability = 0.5; 0.001 ≤ MAF < 0.005; proportion of additive genetic variance explained by the QTL = 0.001; sample size in the test = 1000, 5000; with multiple rare variants simulated as QTL (**c**) and one rare variant simulated as QTL (**d**)

The power values of the methods used to detect rare simulated QTL averaged across 100 replicates are in Figs. 2, 3 and 4 (*p* values adjusted for the effective number of independent SNPs). First, the power values for all rare variant mapping methods across the four MAF classes (0.01 ≤ MAF < 0.02; 0.005 ≤ MAF < 0.01; 0.001 ≤ MAF < 0.005; and MAF < 0.001) were very similar under one of the scenarios. For the scenario with a moderate heritability ($h^{2} = 0.5$), the powers of MONSTER, famBT and famSKAT ranged from 0.19 to 0.42 when multiple rare causal variants were assumed and from 0.09 to 0.30 when one causal rare variant was assumed. Increasing the heritability from 0.3 to 0.8, increased the power to detect QTL from ~0.17 to ~0.61 for MONSTER, famBT and famSKAT when multiple rare causal variants were assumed. No method was able to detect QTL (power ≤ 0.05) that only explained 0.1 % of the genetic variance (Fig. 4c, d). When a QTL explained 0.5 % of the genetic variance, the power increased from ~0.13 to ~0.86 as the number of individuals increased from 1000 to 5000 for MONSTER, famBT and famSKAT (when multiple rare causal variants were assumed) (Fig. 4a, b). However, when the QTL explained only 0.1 % of the genetic variance, there was little increase in power (~0.04 to ~0.15) as the number of individuals increased from 1000 to 5000 (Fig. 4c, d).

When the *p* values of the total number of SNPs are adjusted by Bonferroni correction, DMU_AMAT, EMMAX_AMAT and EMMAX_GMAT had little power (<0.05) in all scenarios (see Additional file 4: Figure S4). However, when the *p* values were adjusted by multiple-testing correction based on the effective number of independent SNPs, DMU_AMAT, EMMAX_AMAT and EMMAX_GMAT had less power in all scenarios compared to the specialized methods for mapping multiple causal rare variants. When only one rare variant contributed to the total QTL variance, i.e. when there was only one variant with a relatively large effect, the powers of the LMM (DMU_AMAT, EMMAX_AMAT and EMMAX_GMAT) were similar compared to the specialized methods for rare variant mapping (MONSTER, famBT and famSKAT) (Figs. 2, 3, 4). With EMMAX, the powers were similar regardless of whether the **A**-matrix or **G**-matrix was used for the kinships (Figs. 2, 3, 4). When heritability increased from 0.3 to 0.8, the power of all methods increased (Figs. 2, 3). In general, the power was greater with multiple rare causal variants than with one causal rare variant across all scenarios for MONSTER, famBT and famSKAT (Figs. 2, 3, 4). With a heritability of 0.5, the power across scenarios with one rare causal variant simulated as a QTL remained similar compared to that across scenarios with multiple rare causal variants simulated as QTL for DMU_AMAT, EMMAX_AMAT and EMMAX_GMAT (Figs. 2, 3, 4) but if the total number of SNPs was adjusted by multiple-testing correction, power increased (see Additional file 4: Figure S4). The power of FamBT, compared to the other methods, was greatest across all scenarios for multiple rare causal variants, while that of famSKAT was highest across most scenarios with only one causal rare variant (Figs. 2, 3, 4).

## Discussion

The objective of our study was to compare the power of several gene-based methods to detect rare variants using simulated phenotype data and imputed whole-genome sequence variants for a bovine population with a complex pedigree structure.

Methods that are specialized for the detection of rare variants in a population of individuals with family relationships (MONSTER, famBT and famSKAT) yielded more power than linear mixed models (DMU_AMAT, EMMAX_AMAT and EMMAX_GAMAT) for the detection of QTL with multiple rare causal variants. The linear mixed model which is the method of choice for association mapping of common variants was less powerful for the detection of QTL with multiple rare causal variants (Figs. 2, 3, 4).

The observed association statistics (*χ*^2^) for data simulated under the null model (no rare variant contributing to the phenotypic variance) followed closely the expected distribution under the null hypothesis for all methods except DMU_AMAT (see Additional file 2: Figure S2). A large number of loci showed a very high observed *χ*^2^ (type I errors) under the null model for DMU_AMAT. This is probably because an extremely low frequency allelic variant will remain confined to a few families or individuals. If, by chance, these families or individuals have extreme phenotypes, that effect will be attributed to the allele resulting in a false positive association. The lower the MAF, the greater the chance that the minor allele is confined to a few families or individuals. Therefore, after filtering out the loci with a very low MAF (MAF < 0.001), the observed *χ*^2^ followed closely the expected *χ*^2^ for DMU_AMAT (see Additional file 2: Figure S2). This result suggests that it is necessary to filter out loci with extremely low MAF when using LMM in order to control false positives. However, this phenomenon was not observed with the EMMAX approach, which could be due to the adjustment of such effects in the first-step of EMMAX when the variance components are estimated.

MONSTER and linear mixed models implemented in DMU_AMAT and EMMAX_AMAT captured most of the total simulated heritability when considering both polygenic variance and the estimated QTL variance (see Additional file 5: Figure S5). DMU_AMAT and EMMAX_AMAT (see Additional file 6: Figure S6) yielded similar estimates of the genetic variance explained by QTL. The genomic heritability estimated by EMMAX_GMAT was considerably lower (0.3) than its simulated value (0.5) (see Additional file 5: Figure S5). The covariance structure among individuals was modeled based on pedigree records for phenotype simulation. The genomic relationships that were estimated from the 50 k SNP data differed considerably from the pedigree-based relationships and therefore explained only part of the additive genetic variance for the trait.

For the simulation with the *ENSBTAG00000035858* gene (see Additional file 7: Figure S7), a similar trend was observed as that found for the *ENSBTAG00000018852* gene (see the "Result" section). The power of detecting QTL with a low MAF with the specialized methods for mapping rare variants was around ~30 % in the scenario with a heritability of 0.5 and where the QTL explained 1 % of the additive genetic variance. Similar results were observed in the simulation with the *ENSBTAG00000035858* gene, i.e. the power of MONSTER, famBT and famSKAT when multiple rare variants explain all the QTL variance was greater (~40 %) than that of the linear mixed models (see Additional file 7: Figure S7). We observed relatively more power for low gene effects and small sample sizes, which is probably because all causal mutations were included in the association analyses. In analyses based on real data, it would be very unlikely that all the causal mutations were included in the SNP sets, for instance because variants may simply be removed during filtration of the data. In our simulation, we also considered the situation with only one rare variant explaining all the QTL variance, and we found that the power of MONSTER, famBT and famSKAT was also greater than that of the linear mixed models when the *p*-values were adjusted by multiple-testing correction for total number of SNPs (see Additional file 4: Figure S4). This was unexpected since rare variant mapping assumes an incorrect architecture for the locus when there is only one causal rare variant. Less power in the LMM analysis for scenarios with a single rare causal variant could result from the association signal being masked under stringent multiple-testing correction. When we used the effective number of independent SNPs to correct for multiple-testing, the powers for scenarios with single causal rare variants were similar to those of other specialized rare variant mapping methods (Figs. 2, 3, 4). However, in GWAS, *p*values are generally adjusted by Bonferroni correction i.e. by dividing the *p* values by the total number of SNPs. However, the false positive rate also increased when the *p* values were not divided by the total number of SNPs (Figs. 2, 3, 4).

Our findings across different scenarios probably reflect the overall power for the detection of rare variants based on QTL variance, genetic architecture and sample size for populations with family relationships as observed in cattle and other livestock species. However, when the QTL effect is small (0.1 % of the additive genetic variance), no method had more than 5 % power (i.e. type I error threshold) for the detection of rare variants with a sample size of 1000 individuals (Fig. 4). As expected, increasing the number of individuals increased the power to detect rare variants with small effects (Fig. 4).

The power of rare variant association mapping methods (MONSTER, famBT and famSKAT) depends on the genetic architecture of the trait because they differ in their assumption about the underlying variants, direction of their effects as well as the correlation structure between rare variants. This was also shown by the simulation on the *ENSBTAG00000035858* gene in the main scenarios (see Additional file 7: Figure S7). Specifically, famBT had the greatest power when multiple rare variants in the test SNP set were simulated as QTL while famSKAT had the greatest power when only one rare variant in the test SNP set was simulated as a QTL. The correlation between rare variants in the test SNP set (*ρ*) was very low when only one rare variant was simulated as the QTL. Therefore, the power of famSKAT (*ρ* = 0) was greatest while that of famBT (*ρ* = 1) was greatest when the statistical method’s assumptions matched the genetic architecture of the trait. However, the differences in power between MONSTER, famBT and famSKAT were very small across all scenarios. Therefore, when applying these methods on real data for mapping rare variants, it is reasonable to consider all three methods since the genetic architecture of the trait under study is usually unknown. In summary, in cattle, it is recommended to use rare variant association mapping methods to identify low frequency genetic variants especially when multiple rare variants are causal and contribute to the trait. Once identified, these rare variants could be exploited for whole-genome prediction of breeding values in the future.

Imputation accuracies of rare variants are lower than those of common variants and this could have a large impact in association analyses for rare variants on real data [42]. We used imputed rare sequence variants in this study instead of simulated genotypes. However, we used simulated phenotypes, assuming that the imputed variants were true. Therefore, imputation errors did not distort the individuals’ phenotypes in our study. By using imputed genotypes, the LD structure and allele frequency spectrum are maintained as observed in our population. Therefore, we expect that using imputed genotypes did not affect the conclusions of our study. In real situations, high-coverage exome sequencing or low-coverage whole-genome sequencing of large number of samples may improve the accuracy of genotype call for the rare variants.

Mutations that change the protein structure or lead to a non-functional protein can have a strong phenotypic impact and may therefore be detectable. However, rare variants with subtle effects may be difficult to identify, even if the sample size is large. Therefore, the gene-based approaches used in our study should be considered for genome-wide mapping of rare variants. Besides, computational cost is an important factor to consider when performing genome-wide rare variant mapping. In our analyses, it took ~11 min to perform rare variant mapping for a sample size of 1000 and ~52 min for a sample size of 5000. Considering that there are ~22,000 annotated genes in the bovine genome, this still implies a huge computational effort when considering all the genes. Therefore, it is important that the algorithms for gene-based mapping are further optimized, but it may also be useful to target rare variants in candidate genes only to save computational time.

## Conclusions

Our findings showed that combining rare variants in a test SNP set with MONSTER, famBT and famSKAT yielded more power to map QTL than linear mixed models for bovine data. We also found that these methods could overcome the confounding of extreme phenotypes in the family mean when mapping rare variants compared to a one-step linear mixed model approach [43]. In fact, linear mixed models were prone to yield large numbers of type I errors for loci with extremely low MAF (MAF < 0.001), while they were not able to correctly detect causal loci with extremely low MAF. However, EMMAX was robust to extremely low MAF. It is recommended to use methods such as the burden test or variance component tests for mapping rare variants in cattle and other livestock with a similar family structure.

## Data availability

The data used in this study originated from the 1000 Bull Genome Project (Daetwyler et al. [30] Nature Genet. 46:858-865). Whole-genome sequence data of individual bulls of the 1000 Bull Genomes Project are already available at NCBI using SRA No. SRP039339 (http://www.ncbi.nlm.nih.gov/bioproject/PRJNA238491).

## Additional files

10.1186/s12711-016-0238-5 Heat map of the relationships between the 5000 sampled bulls.
10.1186/s12711-016-0238-5 Type I error rate for the null models using Bonferroni correction and multiple-testing correction based on the effective number of independent SNPs. (S2a) Type I error rate for the null models using Bonferroni correction. (S2b) Type I error rate for the null models using multiple-testing correction based on the effective number of independent SNPs.
10.1186/s12711-016-0238-5 Quantile–quantile plots for the null models with DMU_AMAT when MAF > 0.001.
10.1186/s12711-016-0238-5 Comparison of mixed linear models with the significance level corrected for effective number of SNPs (p/17) and total number of SNPs (p/635). (S4a and S4b) Heritability = 0.5; 0.01 ≤ MAF < 0.02, 0.005 ≤ MAF < 0.01, 0.001 ≤ MAF < 0.005, MAF < 0.001; proportion of additive genetic variance explained by the QTL = 0.01; sample size in the test = 1000; with multiple rare variants simulated as QTL (a) and one rare variant simulated as a QTL (b). (S4c and S4d) Heritability = 0.3; 0.01 ≤ MAF < 0.02, 0.005 ≤ MAF < 0.01, 0.001 ≤ MAF < 0.005, MAF < 0.001; proportion of additive genetic variance explained by the QTL = 0.01; sample size in the test = 1000; with multiple rare variant simulated as a QTL (c) and one rare variant simulated as a QTL (d). (S4e and S4f) Heritability = 0.8; 0.01 ≤ MAF < 0.02, 0.005 ≤ MAF < 0.01, 0.001 ≤ MAF < 0.005, MAF < 0.001; proportion of additive genetic variance explained by the QTL = 0.01; sample size in the test = 1000; with multiple rare variants simulated as QTL (e) and one rare variant simulated as a QTL (f). (S4 g and S4 h) Heritability = 0.5; 0.001 ≤ MAF < 0.005; proportion of additive genetic variance explained by the QTL = 0.01, 0.005, 0.001; sample size in the test = 1000; with multiple rare variants simulated as a QTL (g) and one rare variant simulated as a QTL (h). (S4i and S4j) Heritability = 0.5; 0.001 ≤ MAF < 0.005; proportion of additive genetic variance explained by the QTL = 0.005; sample size in the test = 1000, 5000; with multiple rare variants simulated as QTL (i) and one rare variant simulated as QTL (j). (S4 k and S4 l) Heritability = 0.5; 0.001 ≤ MAF < 0.005; proportion of additive genetic variance explained by the QTL = 0.001; sample size in the test = 1000, 5000; with multiple rare variants simulated as QTL (k) and one rare variant simulated as QTL (l).
10.1186/s12711-016-0238-5 Computed heritabilities compared across methods.
10.1186/s12711-016-0238-5 Comparison of the variances explained by SNPs between EMMAX_AMAT, EMMAX_GMAT and DMU_AMAT.
10.1186/s12711-016-0238-5 Comparison of the power of different methods in different scenarios for the *ENSBTAG00000035858* gene (*p* values with calculation of independent tests for linear mixed models). (S7a and S7b) Heritability = 0.5; 0.01 ≤ MAF < 0.02, 0.005 ≤ MAF < 0.01, 0.001 ≤ MAF < 0.005, MAF < 0.001; proportion of additive genetic variance explained by the QTL = 0.01; sample size in the test = 1000; with multiple rare variants simulated as QTL (a) and one rare variant simulated as a QTL (b). (S7c and S7d) Heritability = 0.3; 0.01 ≤ MAF < 0.02, 0.005 ≤ MAF < 0.01, 0.001 ≤ MAF < 0.005, MAF < 0.001; proportion of additive genetic variance explained by the QTL = 0.01; sample size in the test = 1000; with multiple rare variants simulated as a QTL (c) and one rare variant simulated as a QTL (d). (S7e and S7f) Heritability = 0.8; 0.01 ≤ MAF < 0.02, 0.005 ≤ MAF < 0.01, 0.001 ≤ MAF < 0.005, MAF < 0.001; proportion of additive genetic variance explained by the QTL = 0.01; sample size in the test = 1000; with multiple rare variants simulated as QTL (e) and one rare variant simulated as a QTL (f).
