# Supplementary figures and images for: Comparison of gene-based rare variant association mapping methods for quantitative traits in a bovine population with complex familial relationships
Source: Genet Sel Evol. 2016 Aug 17;48:60. doi: 10.1186/s12711-016-0238-5 (PMC4989328; doi:10.1186/s12711-016-0238-5)

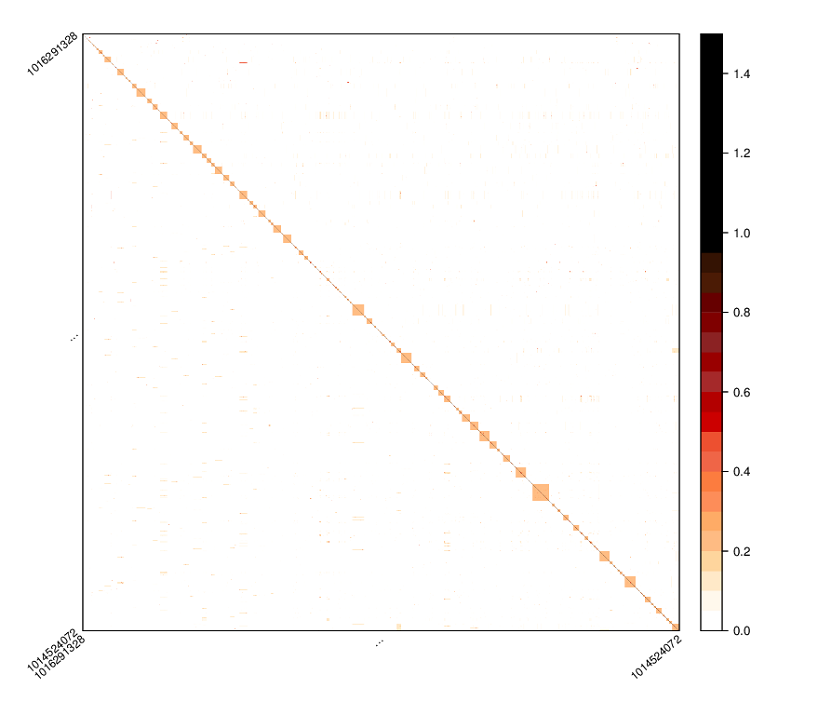

Supplement: Supplementary file 1 — 10.1186/s12711-016-0238-5 Heat map of the relationships between the 5000 sampled bulls. [file 12711_2016_238_MOESM1_ESM.tif]

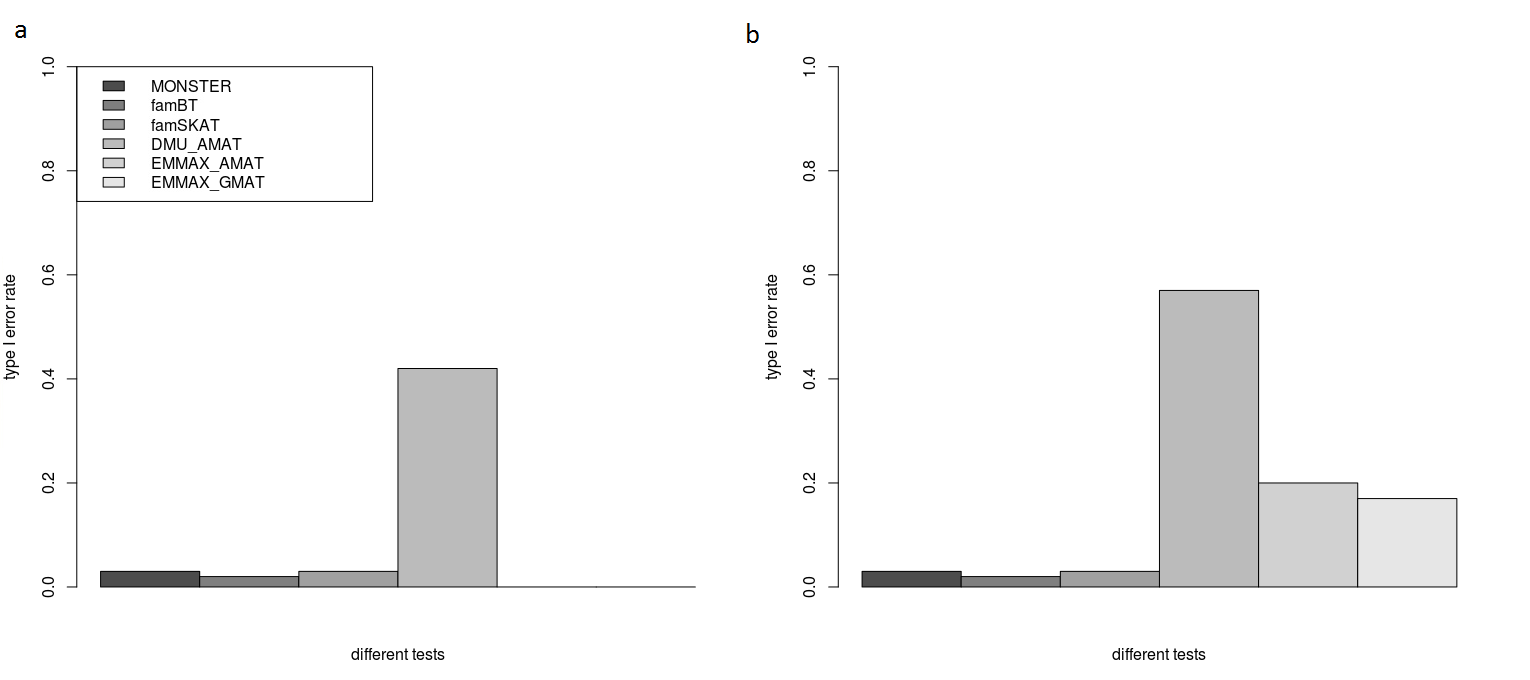

Supplement: Supplementary file 2 — 10.1186/s12711-016-0238-5 Type I error rate for the null models using Bonferroni correction and multiple-testing correction based on the effective number of independent SNPs. (S2a) Type I error rate for the null models using Bonferroni correction. (S2b) Type I error rate for the null models using multiple-testing correction based on the effective number of independent SNPs. [file 12711_2016_238_MOESM2_ESM.tif]

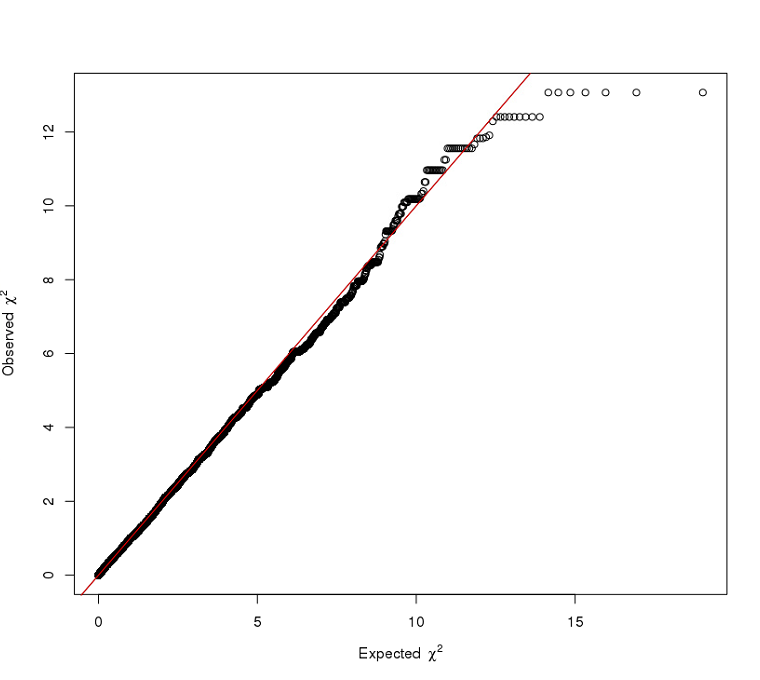

Supplement: Supplementary file 3 — 10.1186/s12711-016-0238-5 Quantile–quantile plots for the null models with DMU_AMAT when MAF > 0.001. [file 12711_2016_238_MOESM3_ESM.tif]

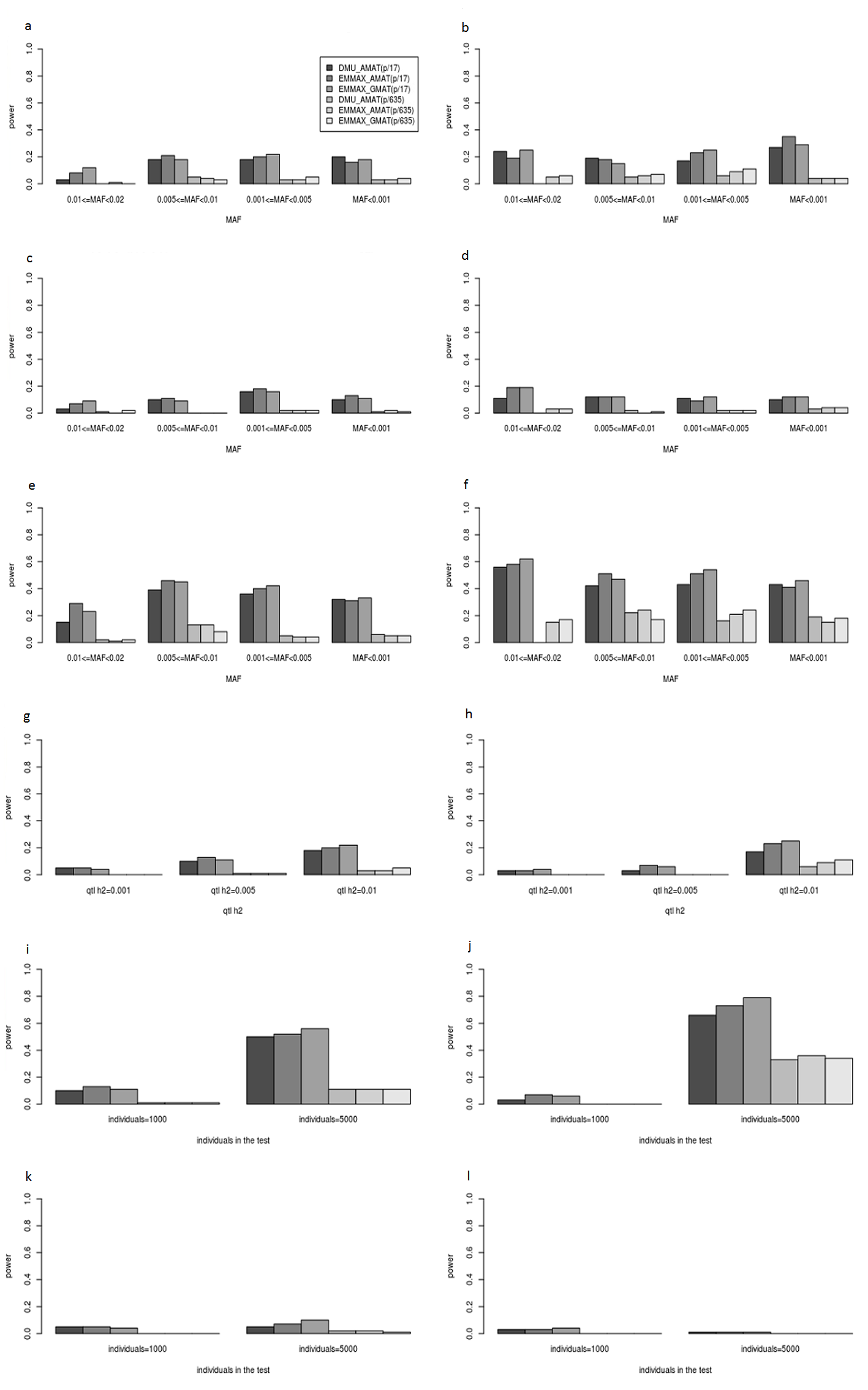

Supplement: Supplementary file 4 — 10.1186/s12711-016-0238-5 Comparison of mixed linear models with the significance level corrected for effective number of SNPs (p/17) and total number of SNPs (p/635). (S4a and S4b) Heritability = 0.5; 0.01 ≤ MAF < 0.02, 0.005 ≤ MAF < 0.01, 0.001 ≤ MAF < 0.005, MAF < 0.001; proportion of additive genetic variance explained by the QTL = 0.01; sample size in the test = 1000; with multiple rare variants simulated as QTL (a) and one rare variant simulated as a QTL (b). (S4c and S4d) Heritability = 0.3; 0.01 ≤ MAF < 0.02, 0.005 ≤ MAF < 0.01, 0.001 ≤ MAF < 0.005, MAF < 0.001; proportion of additive genetic variance explained by the QTL = 0.01; sample size in the test = 1000; with multiple rare variant simulated as a QTL (c) and one rare variant simulated as a QTL (d). (S4e and S4f) Heritability = 0.8; 0.01 ≤ MAF < 0.02, 0.005 ≤ MAF < 0.01, 0.001 ≤ MAF < 0.005, MAF < 0.001; proportion of additive genetic variance explained by the QTL = 0.01; sample size in the test = 1000; with multiple rare variants simulated as QTL (e) and one rare variant simulated as a QTL (f). (S4 g and S4 h) Heritability = 0.5; 0.001 ≤ MAF < 0.005; proportion of additive genetic variance explained by the QTL = 0.01, 0.005, 0.001; sample size in the test = 1000; with multiple rare variants simulated as a QTL (g) and one rare variant simulated as a QTL (h). (S4i and S4j) Heritability = 0.5; 0.001 ≤ MAF < 0.005; proportion of additive genetic variance explained by the QTL = 0.005; sample size in the test = 1000, 5000; with multiple rare variants simulated as QTL (i) and one rare variant simulated as QTL (j). (S4 k and S4 l) Heritability = 0.5; 0.001 ≤ MAF < 0.005; proportion of additive genetic variance explained by the QTL = 0.001; sample size in the test = 1000, 5000; with multiple rare variants simulated as QTL (k) and one rare variant simulated as QTL (l). [file 12711_2016_238_MOESM4_ESM.tif]

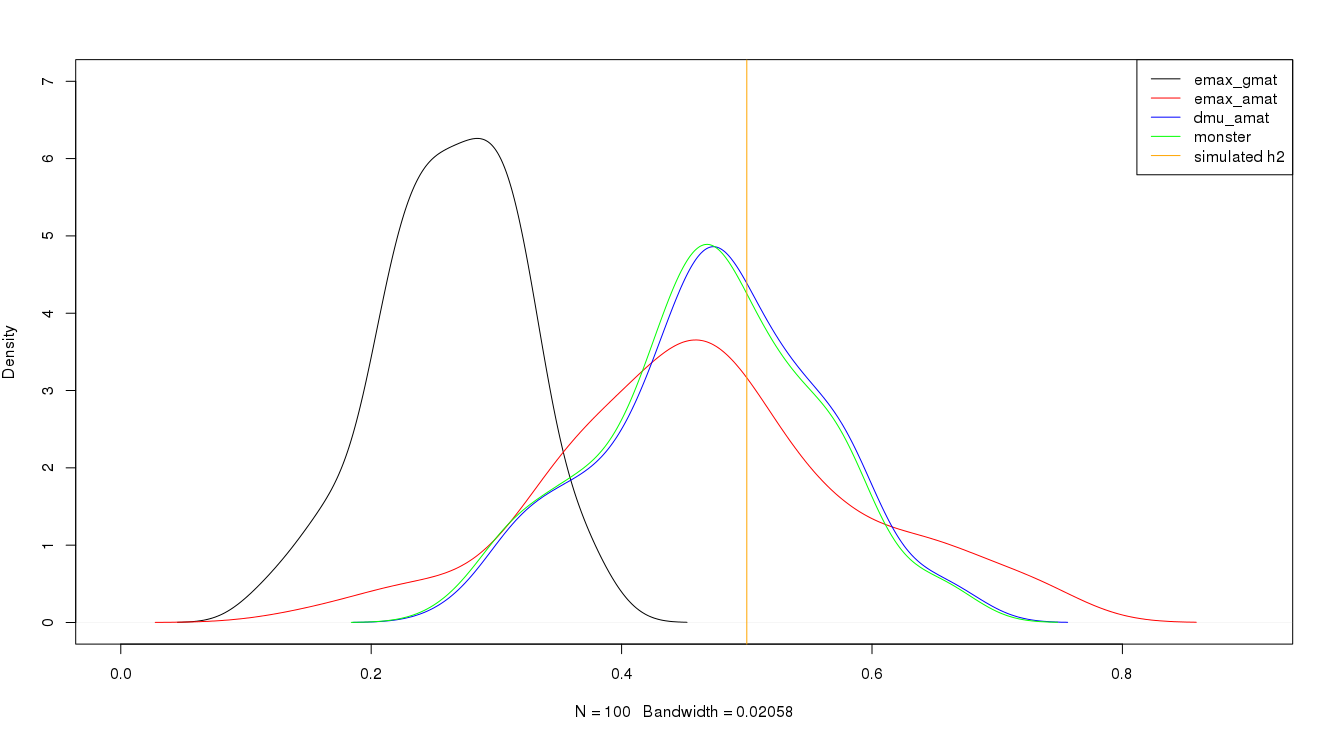

Supplement: Supplementary file 5 — 10.1186/s12711-016-0238-5 Computed heritabilities compared across methods. [file 12711_2016_238_MOESM5_ESM.tif]

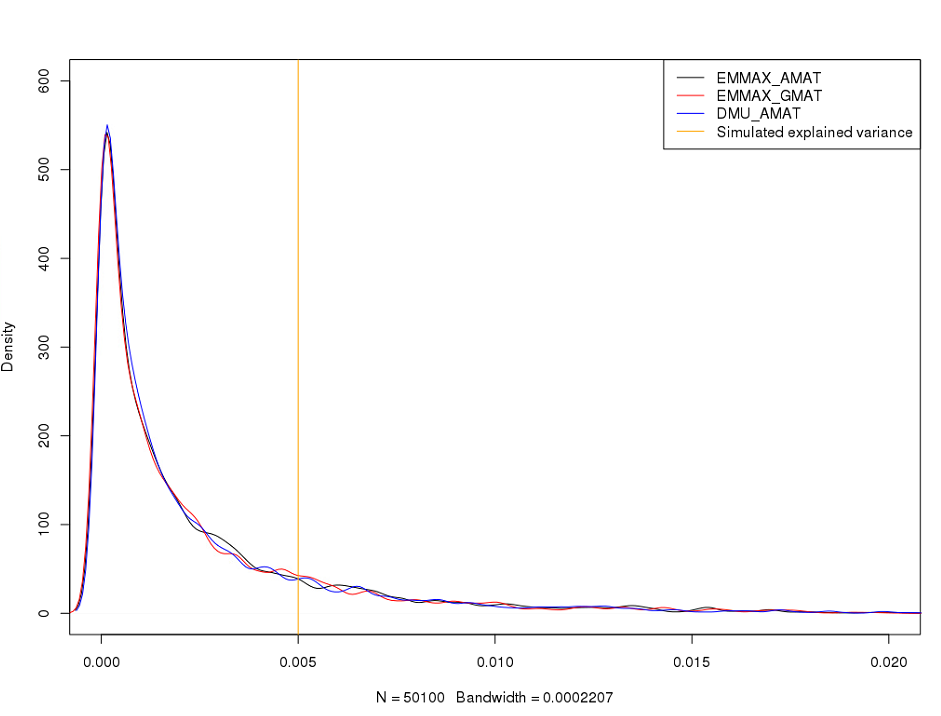

Supplement: Supplementary file 6 — 10.1186/s12711-016-0238-5 Comparison of the variances explained by SNPs between EMMAX_AMAT, EMMAX_GMAT and DMU_AMAT. [file 12711_2016_238_MOESM6_ESM.tif]

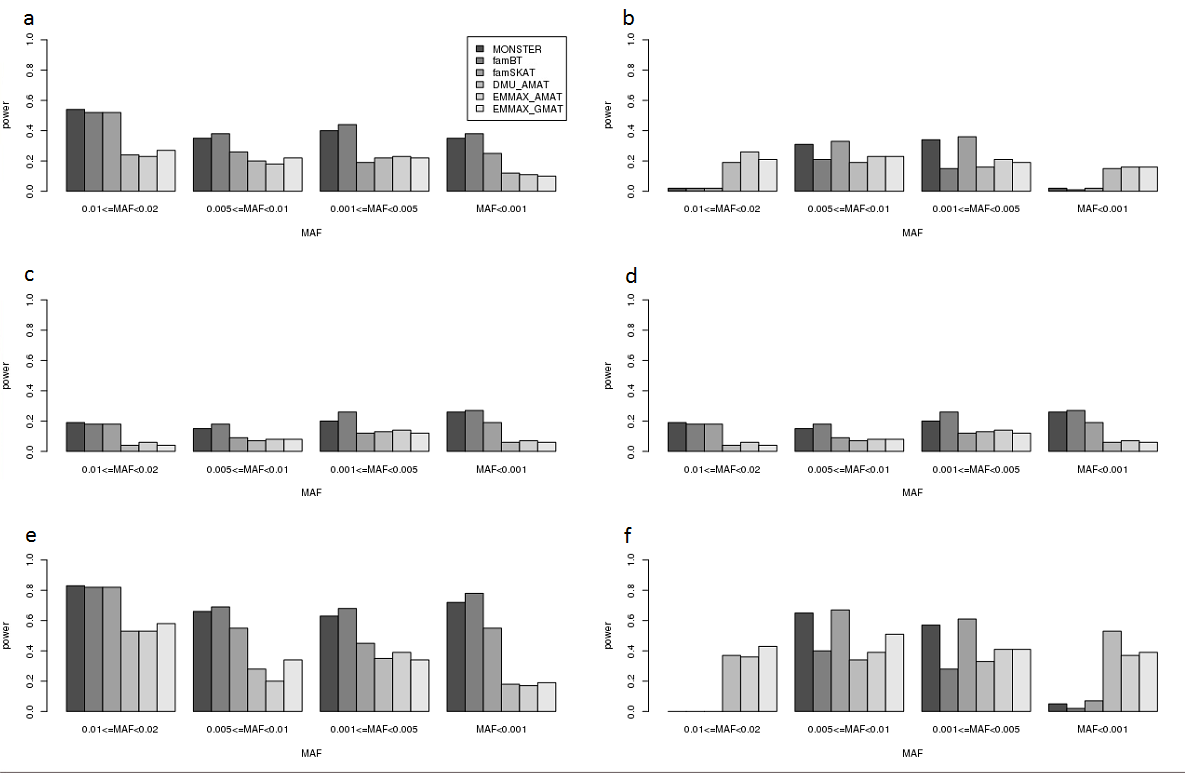

Supplement: Supplementary file 7 — 10.1186/s12711-016-0238-5 Comparison of the power of different methods in different scenarios for the ENSBTAG00000035858 gene (p values with calculation of independent tests for linear mixed models). (S7a and S7b) Heritability = 0.5; 0.01 ≤ MAF < 0.02, 0.005 ≤ MAF < 0.01, 0.001 ≤ MAF < 0.005, MAF < 0.001; proportion of additive genetic variance explained by the QTL = 0.01; sample size in the test = 1000; with multiple rare variants simulated as QTL (a) and one rare variant simulated as a QTL (b). (S7c and S7d) Heritability = 0.3; 0.01 ≤ MAF < 0.02, 0.005 ≤ MAF < 0.01, 0.001 ≤ MAF < 0.005, MAF < 0.001; proportion of additive genetic variance explained by the QTL = 0.01; sample size in the test = 1000; with multiple rare variants simulated as a QTL (c) and one rare variant simulated as a QTL (d). (S7e and S7f) Heritability = 0.8; 0.01 ≤ MAF < 0.02, 0.005 ≤ MAF < 0.01, 0.001 ≤ MAF < 0.005, MAF < 0.001; proportion of additive genetic variance explained by the QTL = 0.01; sample size in the test = 1000; with multiple rare variants simulated as QTL (e) and one rare variant simulated as a QTL (f). [file 12711_2016_238_MOESM7_ESM.tif]
